# A practical flow cytometric approach to leukocyte immunophenotyping in guinea pigs and its potential applications in lymphoid neoplasia

**DOI:** 10.3389/fvets.2026.1926791

**Published:** 2026-10-19

**Authors:** Hana Štěpánová, Edita Jeklová, Miša Škorič, Rea Jarošová, Vladimír Jekl

**Affiliations:** 1 Department of Infectious Diseases and Preventive Medicine, Veterinary Research Institute, Brno, Czechia; 2 Department of Pathological Morphology and Parasitology, Faculty of Veterinary Medicine, University of Veterinary Sciences Brno, Brno, Czechia; 3 Department of Pharmacology and Pharmacy, Faculty of Veterinary Medicine, University of Veterinary Sciences Brno, Brno, Czechia; 4 Jekl & Hauptman Veterinary Clinic – Focused on Exotic Companion Mammal Care, Brno, Czechia

**Keywords:** *Cavia porcellus*, flow cytometry, guinea pig, leukocyte immunophenotyping, lymphoma, lymphoproliferative disorders, neoplasia, veterinary diagnostics

## Abstract

Flow cytometry is an established tool for leukocyte immunophenotyping in human and veterinary medicine, but its use in guinea pigs remains limited. This study aimed to develop a practical flow cytometric methodology for guinea pig leukocyte immunophenotyping, to describe immunophenotypic data from clinically healthy adult guinea pigs, and to assess its diagnostic applicability in selected cases of suspected lymphoproliferative disease. Clinically healthy adult client-owned guinea pigs aged 2–3 years and client-owned guinea pigs examined for presumptive lymphoid neoplasia were analyzed, with an additional experimental cohort included for age-related comparison of the T/B lymphocyte ratio. Multicolor flow cytometry panels combining CD45, MIL-4, panT, CD1b3, CD14, CD4, CD8, and intracellular CD79α were applied to identify major leukocyte populations, including granulocytes, monocytes, T cells, B cells, and CD4/CD8-defined T-cell subsets. In clinically healthy adult guinea pigs, both routine hematology and flow cytometry showed a lymphocyte-dominant peripheral blood profile. Flow cytometry demonstrated that lymphocytes accounted for a median of 62.5% of viable leukocytes, while neutrophils accounted for 8.0%, eosinophils for 8.5%, and monocytes for 3.0%. Within the lymphocyte gate, T cells were the predominant population, representing 78.9% of lymphocytes, while B cells were present in 6.3%; CD8^+^CD4^−^ T cells formed the largest T-cell subset. The T/B lymphocyte ratio did not differ significantly among guinea pigs aged 2 months, 9 months, and 2–3 years. All five histologically confirmed lymphoid neoplasms with a B-cell phenotype were associated with marked B-cell-predominant lymphocytosis in peripheral blood and low T/B ratios. The marked circulating lymphocytosis raised consideration of both lymphoma with peripheral blood involvement and primary B-cell leukemia. In cases in which affected tissue was also examined by flow cytometry, the tissue immunophenotype was consistent with a B-cell lymphoid neoplasm. Overall, the proposed antibody panel is readily implementable for guinea pig leukocyte immunophenotyping, confirms T-cell predominance among peripheral blood lymphocytes in healthy adult guinea pigs. Flow cytometry provided rapid complementary immunophenotypic information in guinea pigs with histologically confirmed lymphoid neoplasia, supporting its use as an adjunct to conventional diagnostic methods.

## 1 Introduction

In veterinary medicine, flow cytometry is widely used for immunophenotyping and diagnosing hematological and immune disorders, but its routine use varies across species. In guinea pigs (*Cavia porcellus*), its clinical application remains limited due to the lack of species-specific antibodies and established diagnostic panels. This is an important limitation, as guinea pigs are both widely used in research and are increasingly encountered in companion animal practice (1, 2). Expanding the use of flow cytometry in this species may therefore contribute to more accurate characterization of immune cell populations and support improved diagnostic and prognostic outcomes.

The gradual development of guinea pig-specific and cross-reactive monoclonal antibodies has provided the basis for immunophenotyping in this species. The introduction of the antibodies in the late 1980s enabled the first immunophenotyping studies in guinea pigs, initially performed using immunohistochemistry (3–6). These developments provided the basis for the first flow cytometric studies, including analysis of the cellular distribution of the guinea pig leukocyte common antigen (7) and characterization of large granular leukocytes unique to guinea pigs, known as Kurloff cells (8, 9). Growing interest in immune profiling led to the development of more comprehensive antibody panels for leukocyte immunophenotyping. These panels have been gradually refined and expanded over time, in parallel with advances in antibody availability and cytometric technologies (10–12). Recent developments based mainly on experimental research include several multi-color flow cytometry panels that allow detailed immunophenotyping of peripheral blood and tissue-associated immune cells, enabling the identification of major leukocyte populations such as monocytes, macrophages, B cells, and T cells in various tissues and blood samples in guinea pigs (13–15).

In general, neoplasia is considered as one of the most common diseases in guinea pigs, particularly in animals older than 4 years. The prevalence of neoplasia increases from 1.4% in animals younger than 6 months to 53.6% in guinea pigs older than 6 years (16). Lymphoproliferative disease has been documented in both spontaneous and experimentally induced cases. Among all organ biopsies examined, the prevalence of lymphoproliferative disorders in two retrospective studies were 7.0 and 10.46% (16, 17). In clinical settings, isolated cases of leukemias, multicentric B-cell lymphoma, disseminated T-cell lymphoma, and epitheliotropic T-cell lymphoma were reported (18–21). Guinea pigs possess an endogenous type C retrovirus, which has been associated with leukemia and exogenous transmission in an inbred strain of guinea pigs; however spontaneous viral-induced leukemia has not been documented in natural settings (22). Clinical signs of lymphoma are typically nonspecific and may include anorexia, lethargy, poor coat condition, peripheral lymphadenopathy, or respiratory distress, depending on the location and extent of the disease (23). Histopathological examination with immunohistochemistry remains the standard diagnostic approach, but immunophenotyping using flow cytometry offers the potential for earlier and more precise classification of lymphoid malignancies. Flow cytometry can also assist in the diagnosis and monitoring of other hematologic or immune-mediated pathological conditions by providing rapid and quantitative assessment of leukocyte populations.

The aim of the present study was to establish a practical flow cytometric panel for guinea pig leukocyte immunophenotyping with direct relevance to veterinary diagnostic practice. Specifically, to provide baseline quantitative peripheral blood data from clinically healthy adult guinea pigs and to demonstrate the applicability of flow cytometry in selected clinical cases, particularly in the evaluation of suspected lymphoproliferative disease.

## 2 Materials and methods

### 2.1 Animals

The study included three cohorts of guinea pigs: clinically healthy client-owned guinea pigs, client-owned clinical patients examined for suspected lymphoma, and guinea pigs from an independent experimental study in which flow cytometry was included for basic immunophenotyping.

Client-owned crossbred guinea pigs (*Cavia porcellus*) were presented to a private veterinary referral clinic for routine health screening or evaluation of clinical disease. Client-owned guinea pigs were examined and sampled as part of routine veterinary diagnostic procedures performed according to best practice veterinary care. Written informed consent for diagnostic procedures, sample collection, and the use of anonymized clinical data was obtained from the owners prior to inclusion in the study. These animals were assigned to a healthy control group (*n* = 13, age 2–3 years) or a presumptive lymphoma group (*n* = 9). Animals were considered healthy when all of the following criteria were met: no previous history of systemic disease, absence of clinical signs of illness at the time of presentation, no abnormalities detected during clinical examination, and no abnormalities were identified on hematological and plasma chemistry examination, fecal parasitology, or urinalysis. Guinea pigs were assigned to the presumptive lymphoma group when at least one of the following criteria was present: generalized peripheral lymphadenopathy, a palpable cervical mass consistent with lymphoid tissue enlargement, and/or leukocytosis detected on hematological examination, predominantly due to an increased lymphocyte count (lymphocytosis). Blood samples were collected from the cranial vena cava as described by Jekl et al. (24). Samples of tissue for histopathological examination were obtained during surgical excisional biopsy of enlarged lymph nodes or cervical masses and for flow cytometry analysis in three animals (clinical case No. 1, 8 and 9). All tissue specimens were submitted for further diagnostic evaluation.

An additional group consisted of crossbred guinea pigs from the untreated control group of an independent animal experiment, which did not receive any experimental intervention. Blood sampling and flow cytometric analysis were performed under a protocol approved by the Expert Commission for Animal Welfare of the Ministry of Agriculture of the Czech Republic. The same animals were sampled and analyzed at 2 and 9 months of age. In this cohort, flow cytometric analysis was limited to T- and B-lymphocyte proportions, and these data were used only to compare the T/B lymphocyte ratio between age categories.

### 2.2 Sample preparation and flow cytometry

Peripheral blood samples were collected into anticoagulant-treated tubes (K₃ EDTA; Aquisel) and processed for flow cytometric analysis. Single-cell suspensions from lymph node samples were prepared by mechanical dissociation of the tissue through a 100-μm cell strainer, followed by rinsing with PBS. For both sample types, ammonium chloride-mediated erythrocyte lysis was performed prior to antibody staining. Three multicolor staining panels and two control panels were designed to identify major guinea pig leukocyte populations, including granulocyte-associated populations, monocytes, T lymphocytes, B lymphocytes, and CD4/CD8-defined T-cell subsets (Table 1). Erythrocyte lysis, washing steps, and antibody staining were performed using the same buffers and staining conditions as reported previously (25, 26).

**Table 1:** Flow cytometry antibody panels used for guinea pig leukocyte analysis.

Panel	Antibodies included	Main purpose
Panel 1	CD45 / MIL-4 / CD14 / panT / CD1b3	CD45-based tissue leukocyte identification; FSC/SSC and MIL-4-based discrimination of granulocytes, monocytes, and lymphocytes
Panel 2	panT / CD1b3 / CD14 / CD4 / CD8	Identification of T and B lymphocytes; CD4/CD8 T-cell subset analysis and CD14-based monocyte discrimination
Panel 3	panT / CD1b3 / CD14 + intracell. CD79α	Confirmation of B-cell identity by intracellular CD79α staining
FMO control for panel 2	CD1b3 / CD14 / CD4 / CD8; panT omitted	FMO control used to support panT gate setting and discrimination of panT-positive/negative populations
Isotype control for panel 3	panT / CD1b3 / CD14 + intracell. IC	Isotype control (IC) for intracellular CD79α staining

The following primary antibodies were used: anti-guinea pig CD45-FITC, clone IH-1, mouse IgG1 (Bio-Rad, MCA1130F); anti-pig CD11R1/MIL-4, clone MIL4, mouse IgG1, purified, cross-reactive with guinea pig (Bio-Rad, MCA1220GA); anti-guinea pig T lymphocytes/panT, clone MsGp7, mouse IgG2a, purified (Bio-Rad, MCA564GA); anti-human CD14-StarBright Violet 610, clone TUK4, mouse IgG2a (Bio-Rad, MCA1568SBV610); anti-guinea pig CD1b3, clone MsGP9, mouse IgG1, either purified or RPE-conjugated (Bio-Rad, MCA566GA and MCA566PE, respectively); anti-guinea pig CD4-RPE, clone CT7, mouse IgG1 (Bio-Rad, MCA749PE); anti-guinea pig CD8-FITC, clone CT6, mouse IgG1 (Bio-Rad, MCA752F); and anti-human CD79α/CD79αcy-RPE, clone HM57, mouse IgG1κ (Agilent/Dako, R7159). Both RPE-conjugated and purified variants of the anti-CD1b3 antibody were used; CD1b3-RPE was included in Panel 1, whereas purified CD1b3 was used in Panel 2 (Table 1). Purified primary antibodies were detected using subclass-specific fluorochrome-conjugated secondary antibodies, namely anti-mouse IgG1-Alexa Fluor 647 (Thermo Fisher Scientific, A21240) and anti-mouse IgG2a-Brilliant Violet 421 (Jackson ImmunoResearch, 115–675-206).

For each sample, 0.5–1 × 10^6^ cells were used in a final volume of 100 μL. The staining buffer consisted of PBS containing 0.2% cold-water fish skin gelatin, 1 g/L NaN₃, and 1.84 g/L Na₄EDTA. Surface staining was performed by incubation with purified primary antibodies followed by the corresponding subclass-specific secondary antibodies. After washing and blocking with 10% mouse serum, directly conjugated antibodies were added. After surface staining, propidium iodide was added, and samples were incubated for 5 min before washing. For intracellular detection of CD79α, cells were fixed and permeabilized using the BD Cytofix/Cytoperm™ Fixation/Permeabilization Kit (BD Biosciences), according to the manufacturer’s instructions. Anti-CD79α-RPE or the corresponding RPE-conjugated isotype control was added during the permeabilization step. After staining, samples were washed and resuspended in staining buffer prior to acquisition.

During panel optimization, fluorescence-minus-one (FMO) controls were performed for all markers included in each staining panel. For routine sample analysis, two controls were retained: an isotype control for intracellular CD79α staining and a panT FMO control, in which the panT primary antibody was omitted while the secondary antibody was retained.

Flow cytometry was performed using a BD LSRFortessa flow cytometer operated with BD FACSDiva software, version 6.0, and data were analyzed using FlowJo software, version 10.6.1. Doublets were excluded by plotting forward scatter area against forward scatter height or width. Viability assessment was performed only in panels analyzed without fixation, where dead cells were excluded based on propidium iodide staining. Detailed representative gating strategies for the individual panels are provided in Supplementary Figures S1–S4. For animals aged 2 and 9 months, flow cytometric analysis was restricted to a reduced antibody panel including panT, CD1b3, and CD14, and only the proportions of T and B lymphocytes were assessed.

### 2.3 Hematological parameters

Total white blood cell counts (WBC) were assessed using the auto hematology analyzer BC-2800Vet (Mindray) or ProCyte Dx (IDEXX). Differential leukocyte counts were determined by counting 200 cells in the blood smears stained with May–Grünwald and Giemsa–Romanowski (Penta Chemicals).

### 2.4 Histopathology and immunohistochemistry

Histopathological examination was performed as part of the diagnostic evaluation of affected tissues and used to characterize the underlying lesions. PAX5 immunohistochemistry was subsequently included to support B-cell lineage assignment in tissue sections, as PAX5 has previously been used as a nuclear B-cell marker for the immunohistochemical identification of B lymphocytes in guinea pig (27).

Tissue samples intended for routine histopathological examination were fixed in 10% neutral buffered formalin, dehydrated, embedded in paraffin wax, sectioned at 4 μm, and stained with hematoxylin and eosin (H&E). Immunohistochemical staining for PAX5 was performed on frozen tissue sections. The slides were rehydrated in PBS and incubated with 0.3% Triton X-100 in PBS. Endogenous enzyme activity was quenched using Dual Endogenous Enzyme-Blocking Reagent (DAKO) for 10 min. After washing, Protein Block (DAKO) was applied for 5 min. The sections were then incubated with monoclonal mouse anti-human PAX5/B-cell-specific activator protein antibody, clone DAK-Pax5 (DAKO), diluted 1:10 in PBS, for 90 min at 37 °C in a humidified chamber. For negative controls, PBS was applied instead of the primary antibody. After washing, the sections were incubated with EnVision reagent (HRP, Mouse; DAKO) for 30 min at 37 °C in a humidified chamber. Immunoreactivity was visualized using the Liquid DAB+ Substrate Chromogen System (DAKO) for approximately 5 min. Finally, the sections were rinsed in distilled water, lightly counterstained with Mayer’s hematoxylin (Penta Chemicals), and mounted in CC/Mount (Diagnostic BioSystems, USA). All washing steps were performed three times for 5 min in PBS containing 0.1% Tween 20.

### 2.5 Statistical analysis

Age-dependent differences in the proportions of T and B lymphocytes were analyzed using a nonparametric Kruskal–Wallis test followed by Dunn’s multiple comparisons test to assess differences between pairs of age groups. *p*-values < 0.05 were considered statistically significant. Statistical analysis was performed using GraphPad Prism software, version 11.0.1 (GraphPad Software).

## 3 Results

### 3.1 Flow cytometry

A series of multicolor flow cytometry panels was established to characterize granulocytes, monocytes, T cells, and B cells in guinea pig peripheral blood and solid tissues. In solid tissue samples, CD45 was particularly important for determining total leukocyte proportions (Supplementary Figure S2), whereas blood samples could be analyzed without this marker. The MIL-4-containing panel enabled differentiation of granulocytes, monocytes, and lymphocytes based on FSC/SSC characteristics together with MIL-4, as previously described by Takizawa et al. (28), and CD14 expression (Table 1, panel 1). Granulocytes were identified as MIL-4^+^ cells and further subdivided according to SSC intensity into MIL-4^+^SSC^med^ neutrophils and MIL-4^+^SSC^hi^ eosinophils (Supplementary Figure S1). Although, to our knowledge, this population has not previously been defined as eosinophils in guinea pigs using this flow cytometric approach, its high-SSC profile was consistent with the scatter characteristics commonly attributed to eosinophils in human blood leukocyte analyses (29). The eosinophil identity was further supported by blood-smear morphology (Figure 1), showing cells with larger and more abundant cytoplasmic granules than neutrophils, which in guinea pigs are historically referred to as pseudoeosinophils because of their eosinophilic granules.

**Figure 1:**
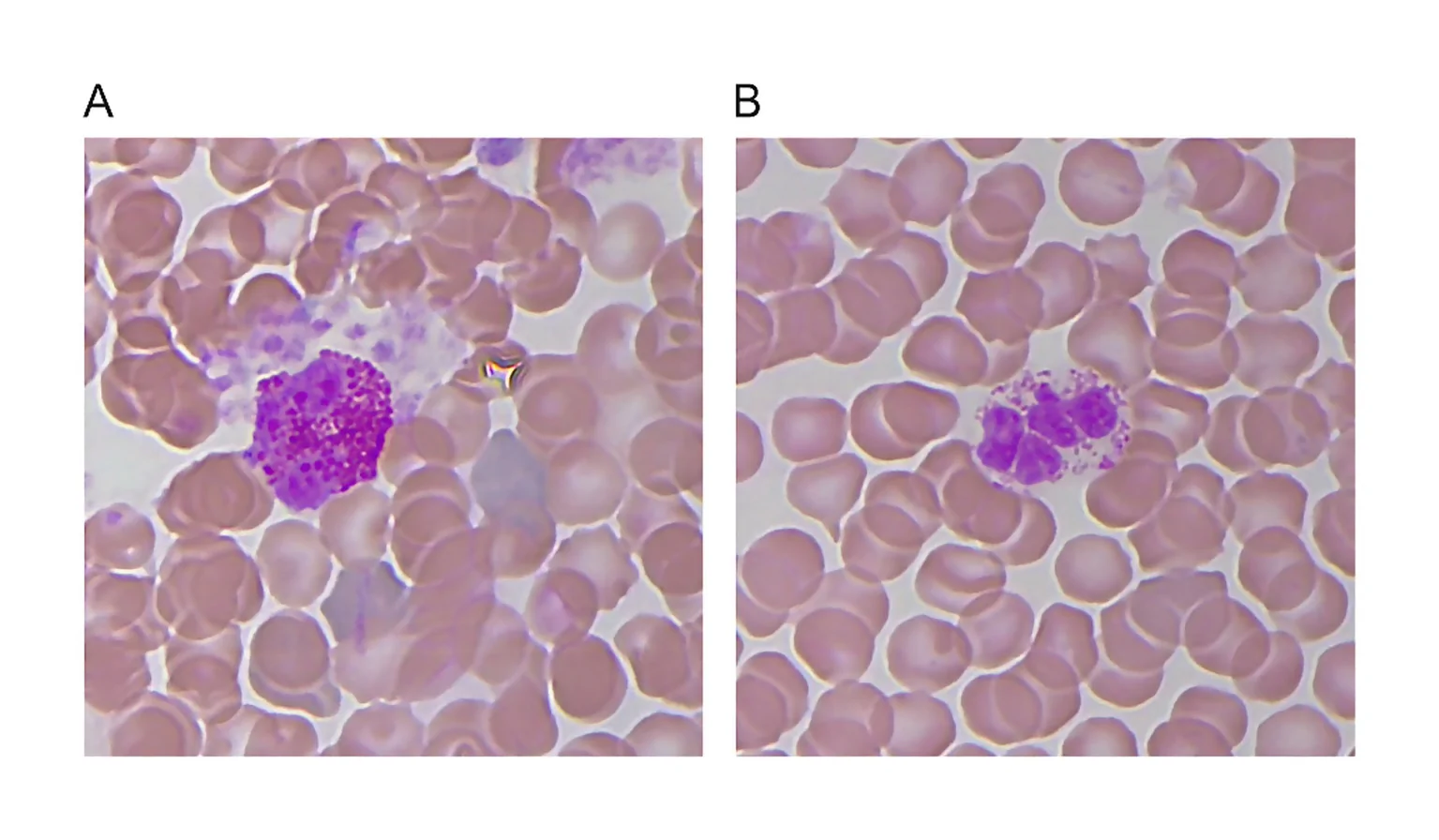
Morphological identification of guinea pig peripheral blood granulocytes. Representative May–Grünwald–Giemsa-stained peripheral blood smears showing granulocyte morphology in guinea pigs. **(A)** Eosinophil with prominent cytoplasmic granules. **(B)** Neutrophil with a segmented nucleus and pale cytoplasmic granulation. Original magnification, ×1,000.

Lymphocytes and monocytes were defined primarily according to FSC/SSC characteristics and MIL-4 expression. Monocytes were identified as MIL-4^+^ and confirmed as CD14^+^ cells with a characteristic FSC/SSC profile (Supplementary Figures S1–S3), whereas the lymphocyte gate was defined as a MIL-4^−^ population with lymphoid FSC/SSC characteristics (Supplementary Figure S1). The lymphocyte population also included panT^−^CD1b3^−^ cells (Supplementary Figure S3), a heterogeneous non-T/non-B population potentially comprising Kurloff cells. Because part of this population extended toward the granulocyte region, its complete interpretation required the MIL-4-containing panel to exclude MIL-4^+^ granulocytes. As MIL-4 was not combined with CD4 and CD8, CD4/CD8 expression was assessed only within the lymphocyte-gated fraction (Supplementary Figure S3); the population was heterogeneous but was not further quantified, as its detailed characterization was beyond the scope of this study.

The principal lymphocyte panel (Table 1, panel 2) enabled detailed lymphocyte immunophenotyping by using CD14 to identify and exclude partially overlapping monocytes (Supplementary Figure S3), panT and CD1b3 to define T and B lymphocytes, and CD4/CD8 expression to further subdivide the T-cells and also non-T/non-B population (Supplementary Figure S3). Because of the relatively low staining index of the panT antibody MsGp7, reliable identification of panT^+^ cells required careful gate adjustment supported by a panT FMO control within the full staining panel, in which the panT primary antibody was omitted while the secondary antibody was retained. Since CD1b3 was not B-cell specific and was also expressed by monocytes, clear discrimination of CD1b3^+^ B cells from monocytes required the use of monocyte-associated markers, particularly CD14 and/or MIL-4 (Supplementary Figures S1–S3). The identity of the B-cell population was therefore confirmed by intracellular staining for the pan-B-cell marker CD79α (Table 1, panel 3), which enabled unequivocal identification of B lymphocytes, as shown in Supplementary Figure S4.

Overall, the established flow cytometry panels enabled reliable discrimination of major leukocyte subsets across sample types and provided a consistent framework for downstream clinical analyses. The following sections present baseline quantitative data obtained from healthy guinea pigs, comparative results across age groups, and selected clinical cases illustrating pathological alterations within distinct immune cell compartments.

### 3.2 Healthy guinea pigs

In healthy adult guinea pigs aged 2–3 years, the median total WBC count was 10.9 × 10^9^/L (min–max, 6.8–13.8 × 10^9^/L). Routine hematological analysis showed that lymphocytes represented the predominant leukocyte population, with a median proportion of 67.8% (55.5–85.5%), followed by neutrophils, 10.5% (5.0–28.0%), eosinophils, 5.3% (0.5–19.5%), and monocytes, 2.3% (0.5–4.0%). Flow cytometric analysis confirmed the predominance of lymphoid cells among viable leukocytes and showed a broadly comparable distribution of the major leukocyte populations (Figure 2). Lymphocytes represented the largest population, accounting for 62.5% (50.5–83.3%) of viable leukocytes. Detailed immunophenotypic analysis further demonstrated that T cells formed the predominant lymphocyte subset, representing 78.9% (44.6–89.1%) of lymphocytes, whereas B cells accounted for 6.3% (1.9–17.1%). Among T cells, CD4^−^CD8^+^ cells were the most abundant subset, accounting for 50.7% (24.4–72.5%). The distribution of the remaining leukocyte and T-cell subsets is shown in Figure 2, together with the key gating steps used for their identification. To assess whether the relative distribution of T and B lymphocytes varied with age, the T/B lymphocyte ratio was compared among guinea pigs aged 2 months, 9 months, and 2–3 years. The median T/B lymphocyte ratio was 15.3 (4.6–30.8) in 2-month-old guinea pigs, 9.7 (4.4–26.4) in 9-month-old guinea pigs, and 9.1 (3.3–27.0) in guinea pigs aged 2–3 years. No significant differences in the T/B lymphocyte ratio were detected among age groups (*p* = 0.2611, Kruskal–Wallis test; Figure 3).

**Figure 2:**
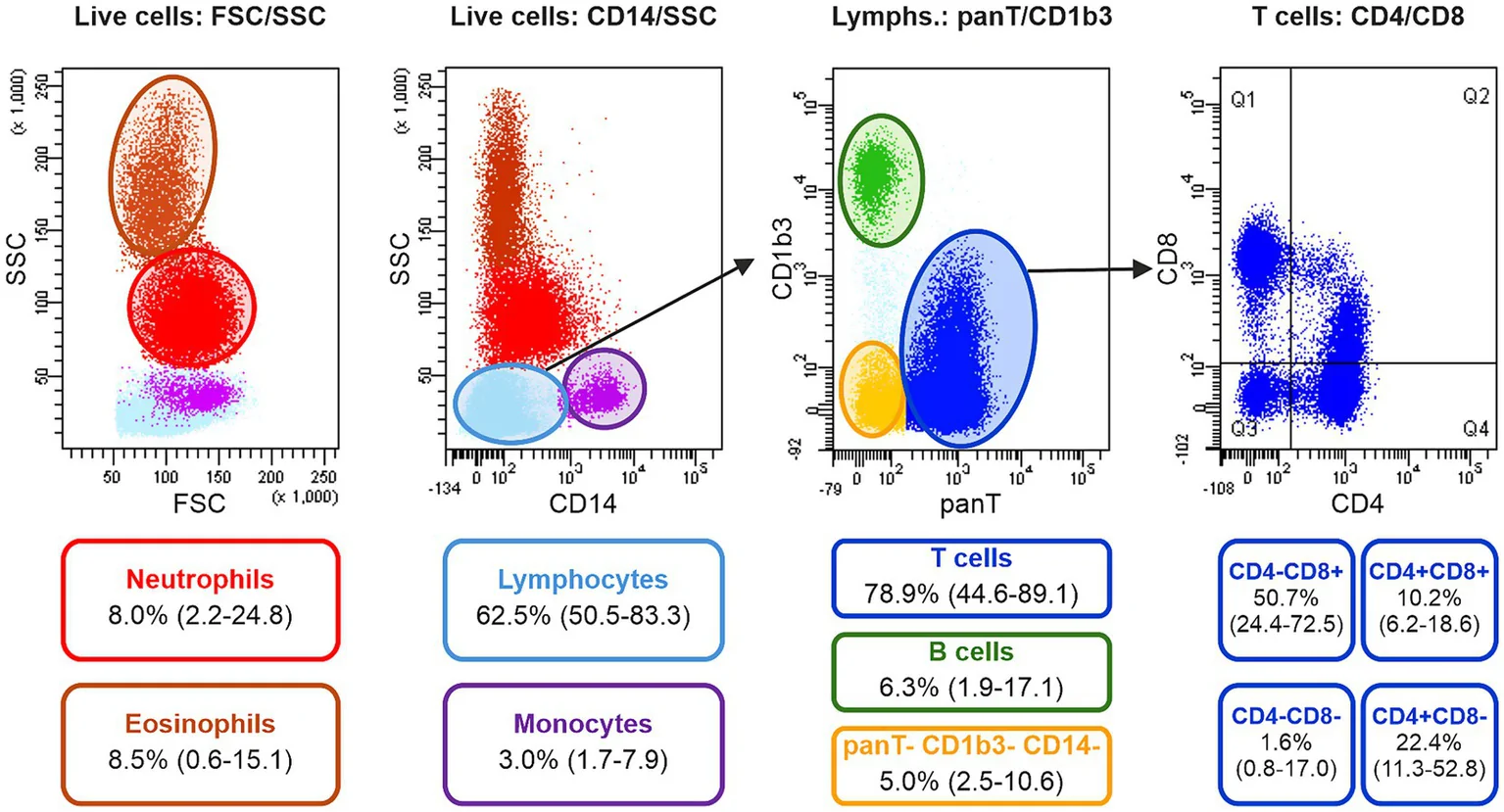
Peripheral blood leukocyte immunophenotyping in clinically healthy adult guinea pigs. Graphical dot plots illustrate the flow cytometric gating strategy used to identify neutrophils, eosinophils, lymphocytes, monocytes, T cells, B cells, panT^−^CD1b3^−^CD14^−^ cells, and CD4/CD8-defined T-cell subsets in peripheral blood. Values are shown as median percentage (min–max). Neutrophils, eosinophils, lymphocytes, and monocytes are expressed as percentages of viable leukocytes; T cells, B cells, and panT^−^CD1b3^−^CD14^−^ cells as percentages of gated lymphocytes; and CD4/CD8-defined subsets as percentages of gated T cells.

**Figure 3:**
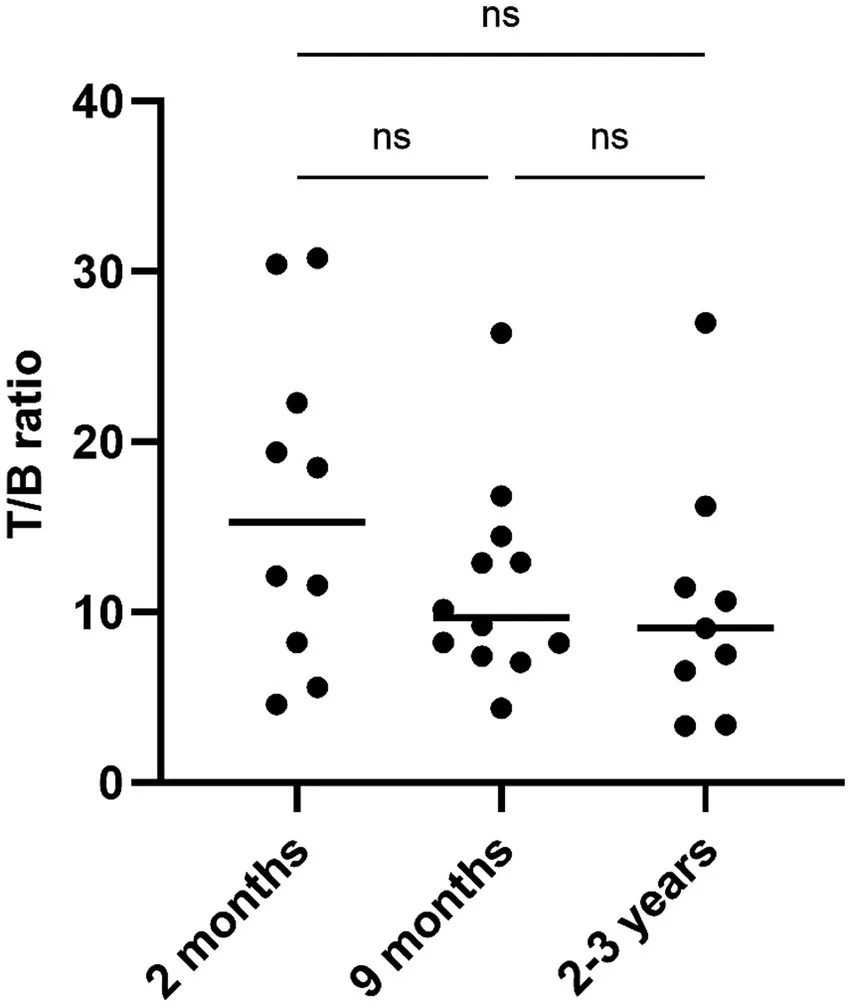
T/B lymphocyte ratio in guinea pigs of different age groups. The graph shows individual T/B lymphocyte ratio values in guinea pigs aged 2 months, 9 months, and 2–3 years. The T/B ratio was calculated as the ratio of the percentage of panT^+^ T cells to the percentage of CD1b3^+^ B cells within the lymphocyte gate. Each dot represents one animal, and horizontal lines indicate median values. No statistically significant differences among age groups were detected using the Kruskal–Wallis test (*p* = 0.2611) followed by Dunn’s multiple comparisons test. ns, not significant.

### 3.3 Clinical cases

According to the inclusion criteria, nine guinea pigs with clinically suspected lymphoproliferative disease were included in the clinical part of the study (Table 2). Peripheral blood flow cytometry was performed in all cases, and tissue flow cytometry was additionally performed in three cases (Nos. 1, 8, and 9). Histopathological examination identified five cases of high-grade lymphomas, two thyroid gland adenocarcinomas, one fibrolipoma, and one case of lymphadenitis. All five cases histologically diagnosed as high-grade lymphoma were associated with marked B-cell-predominant lymphocytosis (62.8–95.3%) in peripheral blood, with low T/B ratios (0.006–0.432). In cases in which affected tissue was also examined by flow cytometry, the tissue immunophenotype demonstrated marked B-cell predominance consistent with a B-cell lymphoid neoplasm. The animals diagnosed with lymphoma had a mean age of 3.7 years and included three females and two males. Cases without histological evidence of lymphoid neoplasia lacked a uniform B-cell-predominant immunophenotypic profile., showing either T-cell predominance or, in the fibrolipoma case, a mixed lymphocyte population. One representative case (Table 2, No. 9), in which flow cytometric analysis was performed both in peripheral blood and affected tissues, is described in detail below and illustrated in Figure 4.

**Table 2:** Clinical, hematological, flow cytometric, and histopathological findings in guinea pigs with suspected lymphoproliferative disease.

Case (No.)	Sex / age (years)	WBC (×10^9^/L)	B cells (%)	T cells (%)	T/B (ratio)	Main clinical presentation	Histopathological diagnosis
1	F / 3.25	167.9	95.3	0.6	0.01	Generalized lymphadenopathy	High-grade lymphoma
2	F / 4.75	55.9	0.5	94.9	189.89	Cervical mass; lymphocytosis	Lymphadenitis
3	F / 4.5	32.6	2.4	93.6	39.01	Cervical mass; lymphocytosis	Thyroid gland adenocarcinoma
4	M / 3.0	139.0	87.6	8.5	0.10	Generalized lymphadenopathy	High-grade lymphoma
5	M / 5.5	36.1	62.8	27.1	0.43	Generalized lymphadenopathy	High-grade lymphoma
6	F / 4.25	228.6	86.6	11.3	0.13	Cervical masses	High-grade lymphoma
7	M / 5.75	16.8	38.2	34.6	0.91	Cervical mass	Fibrolipoma
8	M / 6.0	12.4	11.1	60.3	5.43	Cervical mass	Thyroid gland adenocarcinoma
9	F / 2.5	126.5	86.2	11.0	0.13	Generalized lymphadenopathy	High-grade lymphoma

F, female; M, male; WBC, white blood cell count. B-cell and T-cell percentages and T/B ratios were determined by flow cytometry in peripheral blood.

**Figure 4:**
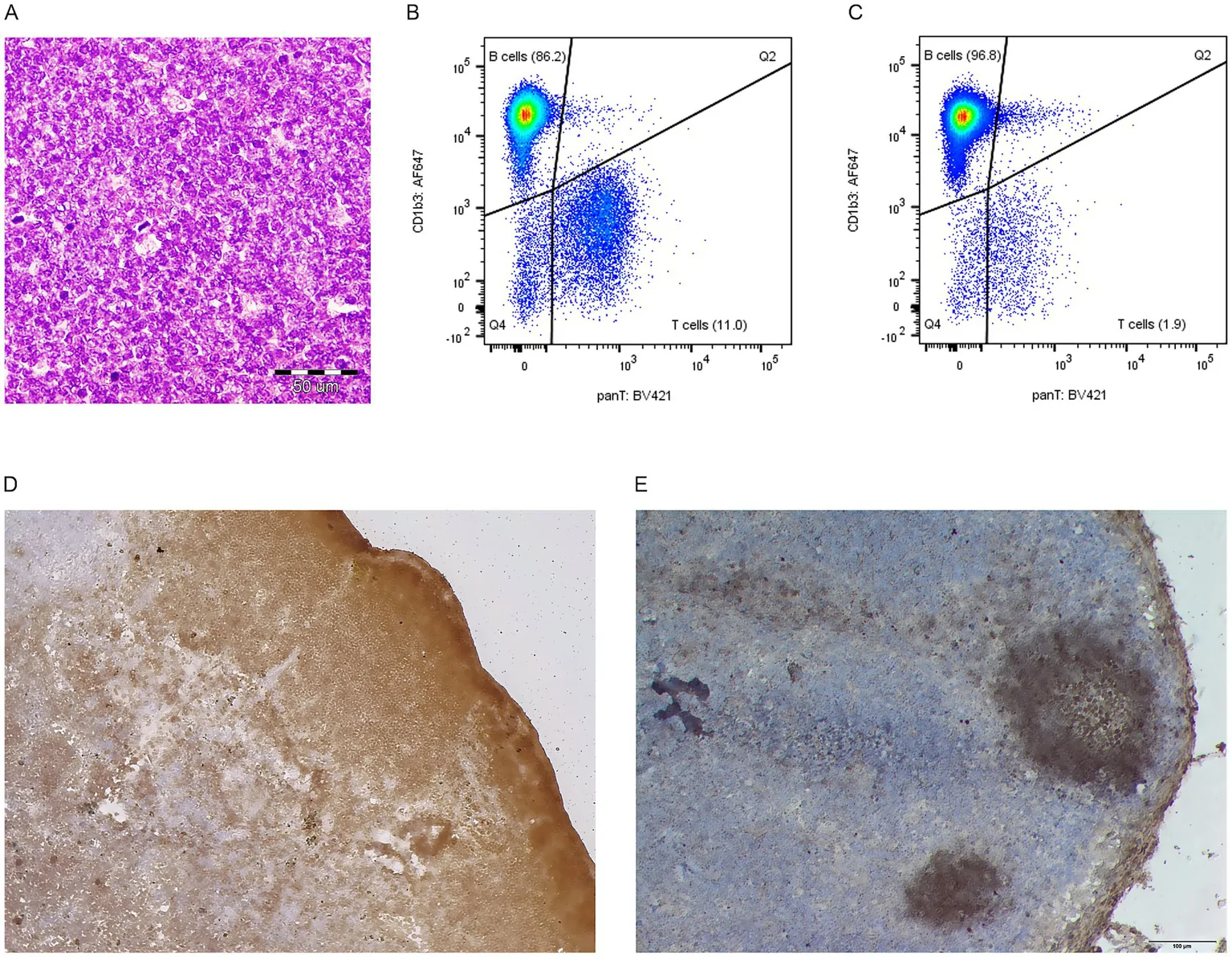
B-cell lymphoma in a guinea pig. Representative findings from a guinea pig presenting with generalized lymphadenopathy and diagnosed with high-grade B-cell lymphoma. **(A)** Histopathological section of the affected submandibular lymph node. H&E stain; original magnification, ×600. **(B,C)** Flow cytometric analysis of peripheral blood **(B)** and submandibular lymph node **(C)**, gated on lymphocytes according to the gating strategy shown in Supplementary Figure S3. The proportions of CD1b3^+^ B cells and panT^+^ T cells are indicated. **(D,E)** Immunohistochemical staining for PAX5. In the affected lymph node, PAX5^+^ neoplastic B cells are diffusely distributed throughout the tissue **(D)**, whereas in a healthy control lymph node, PAX5^+^ B cells are predominantly confined to lymphoid follicles **(E)**; original magnification, ×100.

A 2.5-year-old female guinea pig was presented with apathy and anorexia. Clinical examination and blood analyses revealed generalized lymphadenopathy and marked leukocytosis (WBC 126.5 × 10^9^/L). Due to the animal’s severely compromised clinical condition and metabolic acidosis, euthanasia was performed. Histopathological examination of the submandibular lymph node showed diffuse infiltration by a monomorphic neoplastic lymphoid cell population with cellular atypia, including anisocytosis, anisokaryosis, multiple nucleoli, and mitotic figures, consistent with high-grade lymphoma (Figure 4A). Flow cytometric analysis of peripheral blood showed a marked predominance of lymphocytes (86.8% of viable leukocytes). Within the lymphocyte gate, CD1b3^+^ B cells clearly predominated (86.2%), whereas panT^+^ T cells accounted for 11.0%, resulting in a low T/B ratio of 0.13 (Figure 4B). Given the marked circulating lymphocytosis, a leukemic presentation of the lymphoid neoplasm could not be excluded. The detailed leukocyte distribution in blood was as follows: neutrophils 9.4%, eosinophils 1.2%, lymphocytes 86.8%, and monocytes 1.8%, excluding monocytic expansion. Flow cytometric analysis of the affected submandibular lymph node showed marked B-cell predominance, with 96.8% B cells and 1.9% T cells (Figure 4C). A comparable immunophenotype was also observed in the mesenteric lymph node and spleen, with 91.8% B cells and 6.2% T cells, and 91.8% B cells and 5.7% T cells, respectively (data not shown). These findings were consistent with the histopathological diagnosis and supported a B-cell lineage of the neoplastic lymphoid population. Immunohistochemistry showed diffuse PAX5 positivity of neoplastic cells in the affected lymph node (Figure 4D), whereas in a healthy control lymph node, PAX5^+^ B cells were predominantly confined to lymphoid follicles (Figure 4E). Together with surface CD1b3 and intracellular CD79α positivity detected by flow cytometry, these findings further supported the B-cell lineage assignment of the neoplastic population.

## 4 Discussion

The established three flow cytometry panels provided reproducible identification of major leukocyte lineages in both peripheral blood and solid tissue samples, forming a foundation for subsequent diagnostic evaluation. This methodological framework represents a practical alternative to the more complex multicolor approaches recently described for research applications (14). While such extended protocols maximize analytical resolution, their technical demands and reliance on custom antibody conjugation limit their applicability in diagnostic laboratories. In contrast, our streamlined panel emphasizes robustness, standardization, and ease of implementation—features essential for clinical reliability. The omission of in-house conjugation further reduces hands-on time and minimizes inter-laboratory variability.

A key diagnostic application of flow cytometry in guinea pigs lies in the immunophenotypic classification of lymphomas, particularly in distinguishing B-cell from T-cell origin. Within this context, accurate separation of B cells from monocytes is critical. Both populations bind the anti-CD1b3 antibody (MsGp9) as previously reported (13), with B lymphocytes displaying high (CD1b3hi) and monocytes intermediate (CD1b3med) expression levels. Blocking approaches did not noticeably alter the staining pattern under our conditions, underscoring the importance of including CD14 or MIL-4 for unambiguous monocyte identification. Our work demonstrates that the CD14 clone TUK4 provides reliable cross-reactivity in guinea pigs, offering a practical alternative to the M5E2 clone previously described by Lasco et al. (30).

After exclusion of CD14^+^ monocytes and within the lymphocyte gate, B lymphocytes were identified based on CD1b3 expression, as clone MsGP9 recognizes an antigen expressed by guinea pig B lymphocytes and appears to recognize the guinea pig homologue of CD1b3 (31). This assignment was further supported by intracellular CD79α staining, as CD79α/mb-1 is a component of the B-cell receptor complex and an established marker of normal and neoplastic B cells (32). In the present study, intracellular CD79α was detected using the HM57 clone, which has reported cross-reactivity with multiple animal species, including guinea pigs (33). For T-cell detection, replacement of the CT5 clone, which is no longer commercially available due to loss of the hybridoma, with MsGp7 ensures continuity of diagnostic applicability (15). MsGp7, described by Healey et al. (6), has IgG2a isotype, which facilitates combination with anti-CD1b3 MsGp9 (IgG1) in dual T/B staining panels. This substitution maintains the functionality of existing diagnostic protocols while improving flexibility for indirect staining procedures. Kurloff cells, known to express CT7 (CD4) positivity (8), represent a unique lymphoid subset in guinea pigs. Although they may theoretically contribute to overestimation of CD4^+^ T-cell frequencies, as may other minor populations with lymphocyte-like morphology, their immunophenotypic features (lack of panT marker) allow clear differentiation during diagnostic evaluation, minimizing any clinical ambiguity.

As part of the baseline immunophenotypic characterization of healthy animals, a key finding of the present study was the predominance of lymphocytes in peripheral blood of healthy adult guinea pigs aged 2–3 years. Flow cytometric analysis showed that lymphocytes represented a median of 62.5% of viable leukocytes, which was consistent with routine hematological analysis, where lymphocytes accounted for 67.8% of leukocytes. In contrast, neutrophils consisted of only 8.0% of live cells by flow cytometry and 10.5% by hematology. These findings indicate that peripheral blood leukocytes in healthy adult guinea pigs remain largely dominated by lymphoid populations. Our results, however, differ from the age-associated hematological trends reported in previous studies. Spittler et al. (34) demonstrated that lymphocyte counts decrease with age in Dunkin-Hartley guinea pigs, whereas neutrophils, monocytes, and eosinophils progressively increase. Similarly, Genzer et al. (35) reported an age-related shift from a lymphocyte-dominant to a more neutrophil-dominant leukocyte profile in strain 13/N guinea pigs. Because the guinea pigs included in the present study were approximately 2–3 years old, a relatively greater representation of granulocytes would have been expected based on these reports. Instead, our results revealed a pronounced predominance of lymphoid cells. Several factors may explain this apparent discrepancy, including age, sex, strain, husbandry conditions, and environmental factors, all of which are known to influence hematological parameters (34–36). Studies performed in Dunkin–Hartley and strain 13/N guinea pigs, as well as in other populations, have demonstrated significant differences in leukocyte distributions, suggesting that reference values may not be directly extrapolated between guinea pig colonies or populations (34, 35). Chronic antigenic stimulation or subclinical inflammatory disease may contribute to redistribution of lymphocyte subsets; however, these were ruled out based on history, clinical examination, negative parasitological results, and normal hematology and plasma chemistry analyses. The relatively low proportion of neutrophils observed in the present study may also have contributed to the predominance of the lymphoid compartment. Guinea pig neutrophils are morphologically distinct from neutrophils of many mammalian species and are frequently referred to as pseudoeosinophils because of their eosinophilic cytoplasmic granules (37). Considerable inter-individual variation in neutrophil proportions has been reported in healthy guinea pigs, making interpretation of relative leukocyte percentages challenging (37, 38).

Interestingly, eosinophils accounted for a median of 8.5% of viable leukocytes by flow cytometry and 5.3% by routine hematological analysis, a proportion higher than typically observed in many domestic mammals but consistent with published guinea pig reference data. Both Spittler et al. (34) and Genzer et al. (35) reported relatively broad physiological ranges for eosinophils and demonstrated significant effects of age and sex on eosinophil counts. Consequently, the eosinophil proportion observed in the present study appears to be consistent with normal biological variation within the species.

The immunophenotypic findings further indicate that T cells represent the predominant population within the lymphocyte compartment, accounting for 78.9% of gated lymphoid cells, with CD8^+^ T cells being the largest subset. This T-cell predominance is consistent with previous flow cytometric studies of guinea pig peripheral blood (10, 11, 13). Takizawa et al. (10) reported a more balanced T/B-cell distribution in healthy young Hartley guinea pigs, with T and B cells representing 54.2 and 45.7% of blood lymphocytes, respectively, whereas T-cell predominance was more pronounced in the present cohort. They also reported a higher proportion of CD4^+^ than CD8^+^ lymphocytes, in contrast to our finding that CD4^−^CD8^+^ cells represented the largest T-cell subset. However, direct numerical comparison of CD4/CD8 subsets is limited by differences in the study populations and analytical approaches, including the denominator used for subset reporting. In addition to T and B cells, panT^−^CD1b3^−^CD14^−^ cells represented a median of 5.0% (min–max, 2.5–10.6%) of gated lymphoid cells in the present healthy adult cohort. This non-T/non-B population may include Kurloff cells and other lymphocyte-like populations and may influence the relative percentages of T and B cells within the lymphocyte gate. The T/B lymphocyte ratio therefore provides a more direct comparison between the two major lymphocyte lineages and may serve as a useful complementary diagnostic parameter, particularly in the evaluation of suspected lymphoid neoplasia.

After establishing baseline immunophenotypic data in healthy guinea pigs, the diagnostic applicability of the panels was evaluated in clinical cases of suspected lymphoproliferative disease. Spontaneous lymphoma in guinea pigs represents a heterogeneous group of neoplasms analogous to those described in human and canine patients, where immunophenotypic classification is essential because of substantial differences in biological behavior, prognosis, and therapeutic response among lymphoma subtypes (21, 27). Despite increasing recognition of lymphoma as a clinically important neoplasm in guinea pigs, information on its immunophenotypic distribution remains limited (39). In a retrospective study of spontaneous tumors diagnosed from biopsy and necropsy samples, Nespor et al. (17) reported a lymphoma prevalence of approximately 10%, with T-cell lymphoma representing the predominant subtype. Similarly, Bertram et al. (16) identified lymphoma or leukemia as the most common neoplastic diagnosis (7.0%) among 2,474 guinea pigs examined at necropsy; however, immunophenotypic characterization was not performed. Similarly to other studies, in the present study lymphoma was found in middle-aged and older guinea pigs (average age 3.7 years).

Recent reports have further expanded the recognized clinical spectrum of lymphoma in guinea pigs, including disseminated, ocular, otic, and cutaneous T-cell epitheliotropic forms, highlighting the biological diversity of lymphoid neoplasms in this species (19–21, 40, 41). Such variability underscores the need for reliable methods to distinguish lymphoma subtypes *in vivo*. Evans et al. (27) previously demonstrated the diagnostic utility of immunocytochemistry for differentiating B- and T-cell lymphoma in guinea pigs, providing evidence that immunophenotypic characterization is feasible in this species. Nevertheless, rapid and minimally invasive diagnostic approaches remain poorly investigated. In the present study, flow cytometric immunophenotyping demonstrated a B-cell phenotype in five of nine guinea pigs with clinical findings suggestive of lymphoproliferative disease. The marked peripheral lymphocytosis observed in several affected guinea pigs raised the possibility of a leukemic presentation of the lymphoid neoplasm and therefore warranted consideration of primary leukemia in addition to lymphoma with peripheral blood involvement. In these animals, both a leukemic phase of B-cell lymphoma and primary B-cell leukemia, including B-lymphoblastic leukemia, were considered as differential diagnoses. Histopathological examination subsequently confirmed the presence of lymphoid neoplasia in all cases; however, the available diagnostic data did not allow definitive differentiation between lymphoma with a leukemic phase and primary leukemia in animals with marked circulating lymphocytosis. Furthermore, these results demonstrate that flow cytometry can serve as a valuable adjunct to cytological and histopathological examination and enable rapid immunophenotypic classification of lymphoid neoplasms. The clinical value of immunophenotypic classification has been well established in human and canine oncology (42), where it contributes substantially to the assessment of prognosis and treatment planning. The successful application of flow cytometry in the present study therefore establishes a foundation for future investigations evaluating the biological behavior, prognostic significance, and treatment responsiveness of specific lymphoid neoplasms in guinea pigs.

Limitations of the present study include that flow cytometric analysis was performed primarily on peripheral blood samples obtained from guinea pigs with presumptive lymphoproliferative disease and compared with samples from clinically healthy animals, and the immunophenotypic findings may not fully reflect the cellular composition of the neoplastic tissue itself. Peripheral blood lymphocyte populations may differ from those present in affected lymph nodes, potentially limiting the interpretation of systemic immunophenotypic alterations associated with lymphoid neoplasia. In addition, flow cytometric evaluation of tissue samples was performed in only three cases, limiting comparison of peripheral blood and tissue immunophenotypes. Bone marrow examination was not performed, which further limited the ability to distinguish a leukemic phase of lymphoma from primary leukemia in animals with circulating neoplastic lymphoid cells. Therefore, future studies incorporating larger cohorts and routine flow cytometric analysis of both peripheral blood and affected tissues are warranted to further validate the diagnostic utility of this approach in guinea pigs with lymphoproliferative disorders.

In conclusion, this study describes and applies a practical flow cytometric approach for the immunophenotyping of guinea pig leukocytes in peripheral blood and, in selected cases, tissue-derived single-cell suspensions. In clinically healthy adult guinea pigs aged 2–3 years, both routine hematology and flow cytometry demonstrated a lymphocyte-dominant peripheral blood profile, with T cells representing the predominant lymphocyte population. Together, these data provide baseline immunophenotypic information for clinically healthy adult guinea pigs and form a basis for future studies to establish age- and population-specific reference intervals. In clinical cases, the panel enabled immunophenotypic characterization of histologically confirmed lymphoid neoplasms, supporting flow cytometry as a useful adjunct to cytology, histopathology, and immunohistochemistry in guinea pig lymphoproliferative disease diagnostics.

## Data Availability

The original contributions presented in the study are included in the article/Supplementary material, further inquiries can be directed to the corresponding author/s.

## Supplementary material

SUPPLEMENTARY FIGURE S1 Gating strategy used for leukocyte subset identification in panel 1. Representative flow cytometric plots showing the sequential gating strategy used for identification of major leukocyte populations in peripheral blood. Single cells were first selected based on FSC-A/FSC-H characteristics, followed by exclusion of dead cells as propidium iodide (PI)-positive events to obtain the live-cell population. Among live cells, leukocyte subsets were identified according to MIL-4 expression and SSC properties as lymphocytes (MIL-4^−^/SSC-A^low^), monocytes (MIL-4^+^/SSC-A^low^), and granulocytes (MIL-4^+^/SSC-A^med^/^high^). The granulocyte gate was subsequently subdivided according to FSC/SSC characteristics into eosinophils (SSC-A^high^) and neutrophils (SSC-A^med^). FSC, forward scatter; SSC, side scatter; PI, propidium iodide.

SUPPLEMENTARY FIGURE S2 Gating strategy used for CD45^+^ leukocyte identification in panel 1. Representative flow cytometric plots showing the gating strategy used for identification of CD45^+^ leukocytes in a tissue sample diagnosed as adenocarcinoma. Live cells were first selected by exclusion of dead cells as propidium iodide (PI)-positive events to obtain the live-cell population. Among live cells, CD45^+^ leukocytes were subsequently identified based on CD45 expression. FSC, forward scatter; PI, propidium iodide.

SUPPLEMENTARY FIGURE S3 Gating strategy used for lymphocyte subset identification in panel 2. Representative flow cytometric plots showing the gating strategy used for identification and further characterization of lymphocyte populations in peripheral blood. Live cells were first selected by exclusion of dead cells as propidium iodide (PI)-positive events. From the live-cell population, a combined lymphocyte/monocyte gate was defined according to FSC/SSC characteristics, and monocytes were subsequently separated based on CD14 expression. Within the lymphocyte population, B cells were identified as panT^−^CD1b3^+^ cells, T cells as panT^+^CD1b3^−^ cells, and the remaining panT^−^CD1b3^−^ lymphocytes as non-T/non-B cells. T cells and non-T/non-B cells were further characterized according to CD4 and CD8 expression. FSC, forward scatter; SSC, side scatter; PI, propidium iodide.

SUPPLEMENTARY FIGURE S4 Intracellular CD79α staining used for confirmation of B-cell identity in panel 3. Representative flow cytometric plots showing intracellular CD79α staining within the lymphocyte gate in peripheral blood. The CD1b3^+^ lymphocyte population was evaluated for intracellular CD79α expression, supporting its assignment to the B-cell lineage. The corresponding isotype control is shown for comparison against CD1b3 staining. Propidium iodide (PI) staining was not included in this panel.
